# Supplementary material for: Decoding reward–curiosity conflict in decision-making from irrational behaviors
Source: Nat Comput Sci. 2023 May 15;3(5):418–32. doi: 10.1038/s43588-023-00439-w (PMC10768639; doi:10.1038/s43588-023-00439-w)
Supplement: Supplementary file 1 — Supplementary Figs. 1–8. [file 43588_2023_439_MOESM1_ESM.pdf]

nature computational science

Article

https://doi.org/10.1038/s43588-023-00439-w

Decoding reward–curiosity conflict in decision-making from irrational behaviors

In the format provided by the authors and unedited

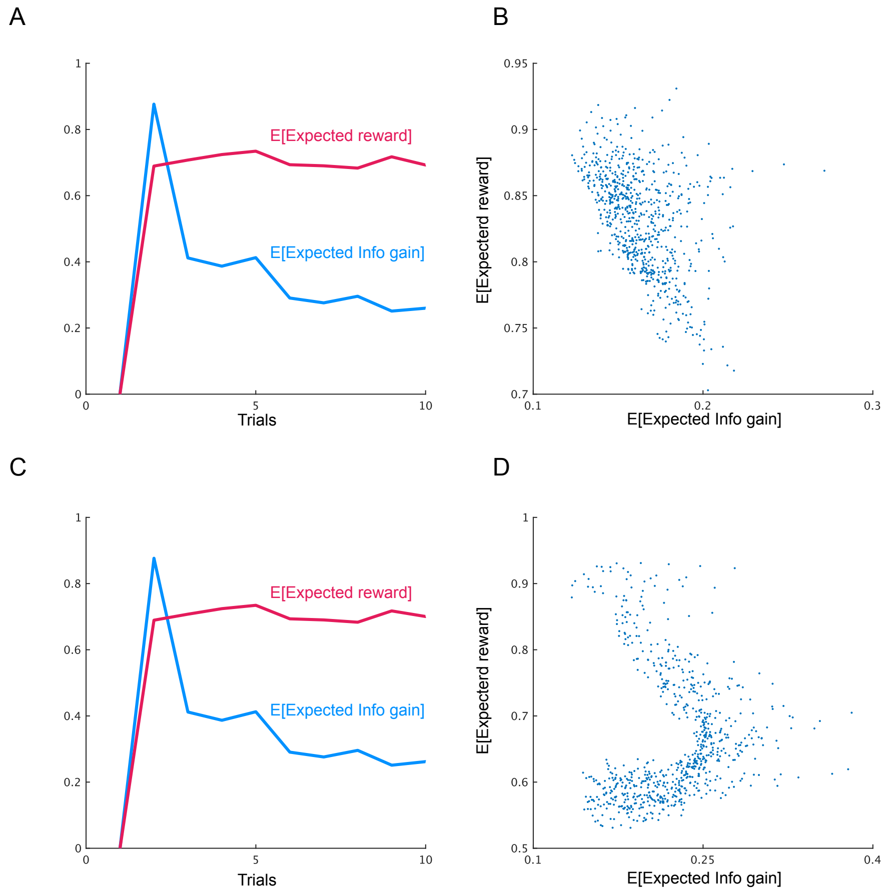

Supplementary Fig. 1: Switching and tread-off between the expected reward and information gain

(A, C) Switching dynamics between the expected reward and information gain in Fig. 2E and F (A), and Fig. 2L and M (C). These values were computed expectations of the expected reward and information gain, respective to action probability. **(B, D)** Relationship between the expectations of the expected reward and information gain in Fig. 2A-G (A), and Fig. 2H-N. Each dot corresponds to each trial, but the first quarter trials were removed ($n = 751$).

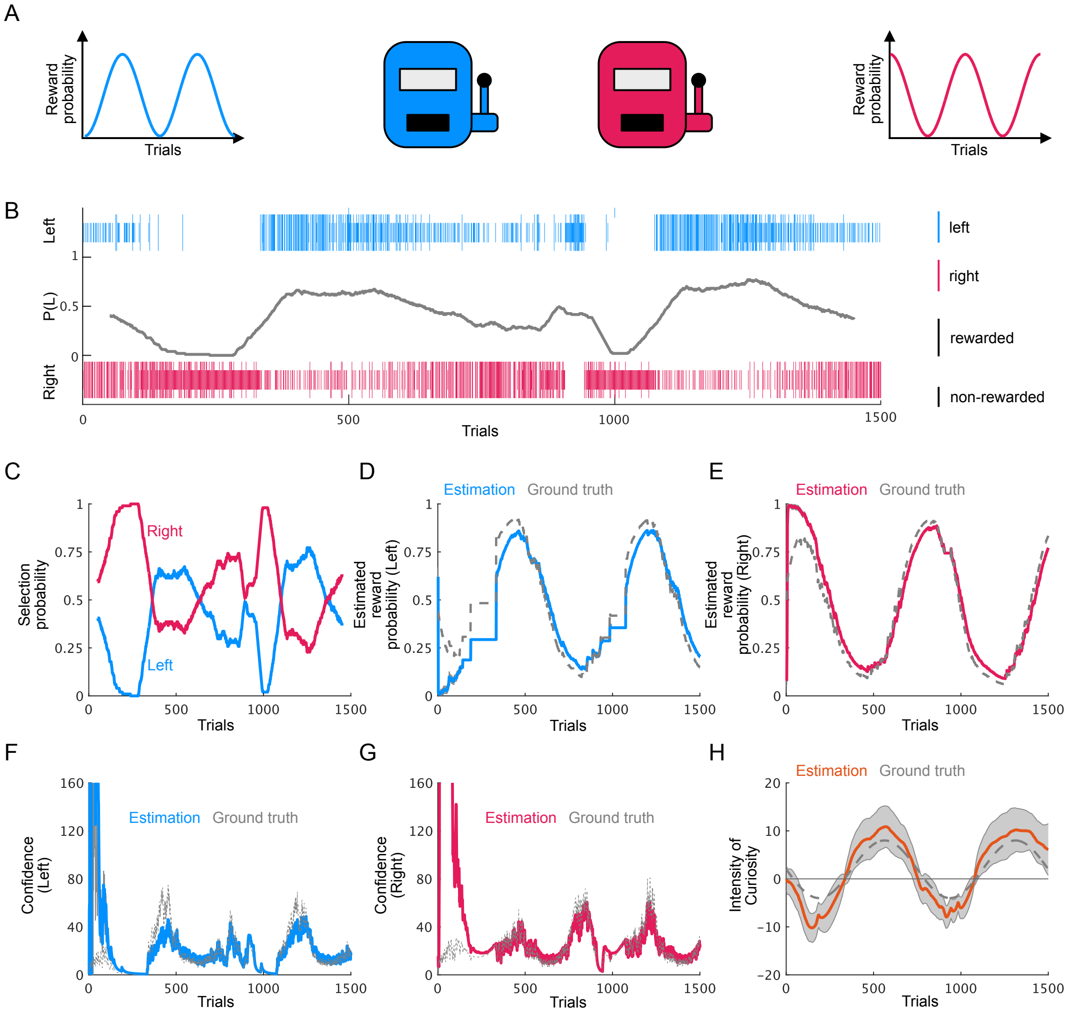

Supplementary Fig. 2: Estimation of the simulated agent's internal state by inverse FEP

(A) Two-choice task with temporally varying reward probabilities and agent having temporally varying curiosity. **(B)** Simulated agent's behaviors. Vertical lines indicate selections of left and right options, respectively. The time-series indicates the moving average of selection probability of the left option with 101 window width. **(C)** Moving average of selection probabilities for left and right options. **(D–H)** Simulated agent's behavior-driven estimations of agent-recognized reward probabilities for left (D) and right (E) options, agent's confidence about recognized reward probabilities for left (F) and right (G) options, and agent's curiosity (H). In this simulation, parameter values were $P_o = 0.8$, $\alpha = 0.05$, $\beta = 2$, and $\sigma = 0.2$. Number of particles was 100,000 in the particle filter. Continuous shaded error bars represent standard deviations for all particles (D-H).

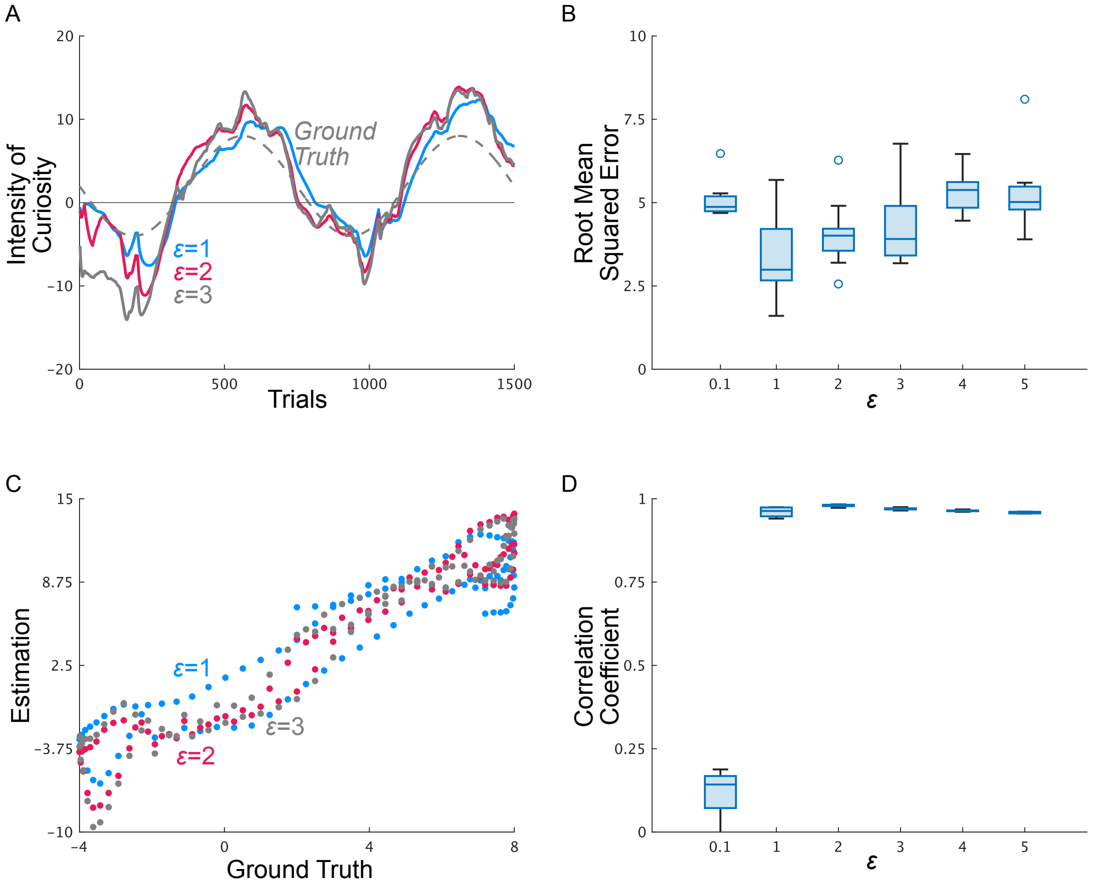

Supplementary Fig. 3: Performance of iFEP depending on ϵ

(A) The estimated curiosity depending on three values of ϵ. The curiosity was estimated by the iFEP from the artificial data generated by the ReCU model. **(B)** Root squared errors between the ground truth and estimation of the curiosity depending on various ϵ. The estimations were repeated by 10 times with different random seeds. **(C)** The relationship of curiosity between the ground truth and estimation depending on ϵ. For this plot, trials were selected every 10 intervals from 500 to 1,500 trials ($n = 101$ for each ϵ). **(D)** Correlation coefficients between the ground truth and estimation of the curiosity depending on various ϵ. The estimations were repeated by 10 times with different random seeds. For all estimations (A-D), number of particles was 100,000 in the particle filter. In box plots (B, D), the central line indicates the median, edges are the lower and upper quantiles, and the upper and lower whiskers represents the highest and lowest values after excluding outliers.

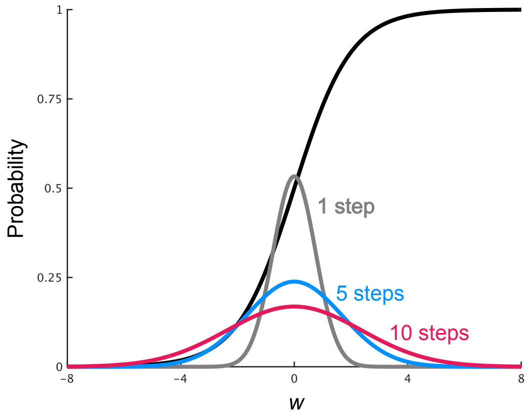

Supplementary Fig. 4: Blurring recognition over time without observation

Recognition of reward probability for a specific option is getting blurred when the agent selects the other option, because the agent assumed random walk of w. The sigmoidal function represents reward probability depending on w. Three Gaussian distributions represent blurring recognition of reward probability after 1, 5, and 10 trials starting with $w_0 = 0$. The stranded deviation increases with trials as $\sqrt{\sigma^2 t}$. Because σ^2 was estimated as $\sigma^2 = 0.560$ from the rat behavioral data by iFEP, the estimated reward probability significantly can change as from 0.5 to 0.5±0.4 during only 10 trials.

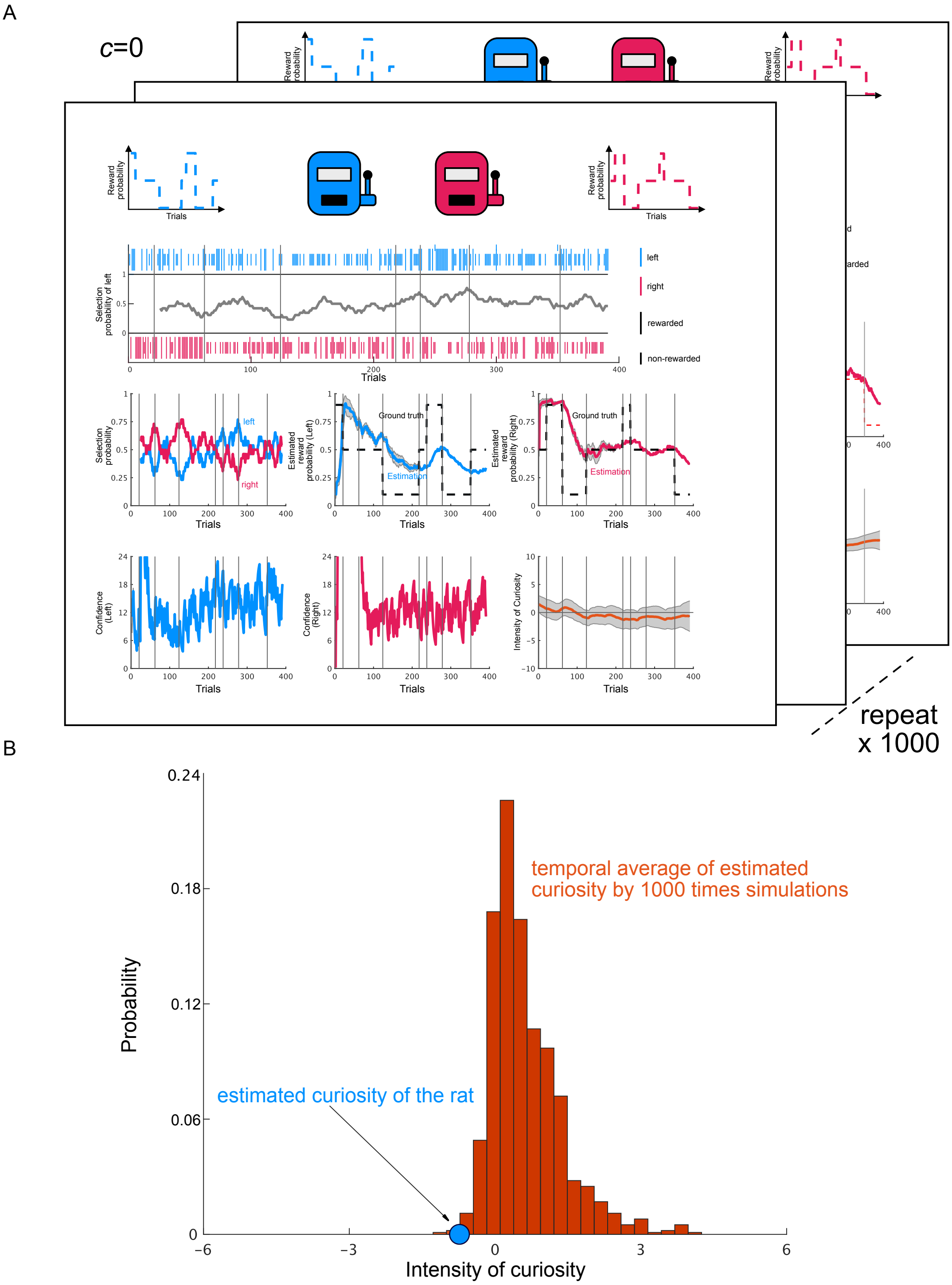

Supplementary Fig. 5: Monte Carlo statistical testing of estimated negative curiosity in rat
(A) Monte Carlo statistical test. Model simulations in the same experimental conditions as in Fig. 5 under a null hypothesis that the rat have no curiosity. Following the null hypothesis, a series of the agent's choices is simulated using iFEP-estimated reward probability recognition and its confidence with zero curiosity (i.e., $c_t = 0$). The same simulations were repeated 1,000 times, and the curiosity was estimated using iFEP for each. Number of particles was 100,000 in the particle filter. **(B)** Null distribution for temporal average of estimated curiosity by 1,000 times simulations. Compared to the null distribution, the temporal average curiosity estimated from the actual rat behavior was located to the left of the significance level (p=0.003, left-sided).

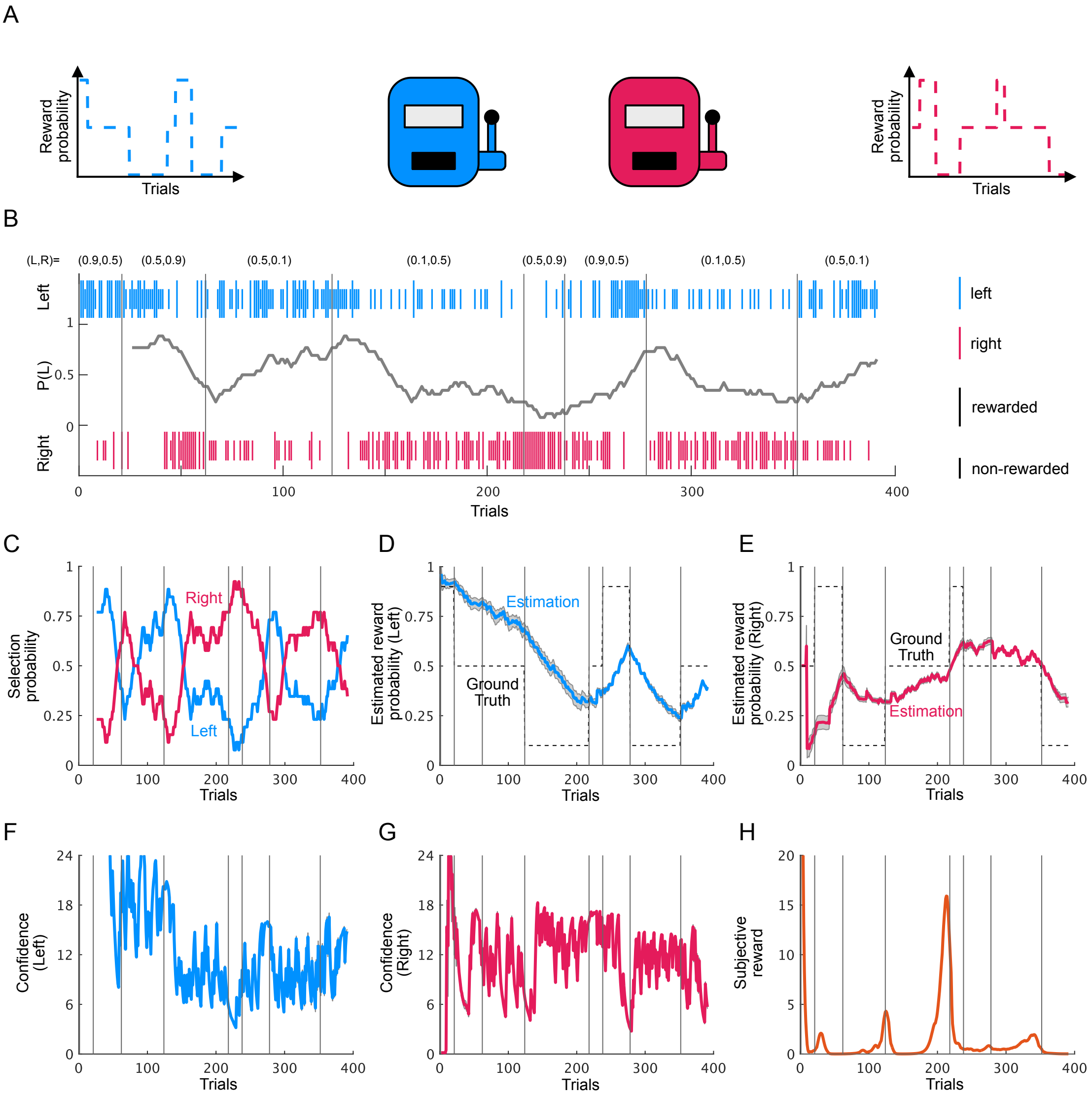

Supplementary Fig. 6: Estimation of the rat's subjective reward by inverse FEP

(A-G) The same as Fig. 5. **(H)** The subjective reward meta-parameter estimated by iFEP with alternative expected net utility ($U(a_{t+1}) = d_t \cdot E[R_{t+1}] + E[Info_{t+1}]$). Estimated parameter values were $\alpha = 0.051$, $\beta = 3.585$, and $\sigma^2 = 0.360$. Number of particles was 100,000 in the particle filter. Continuous shaded error bars represent standard deviations for all particles (D-G).

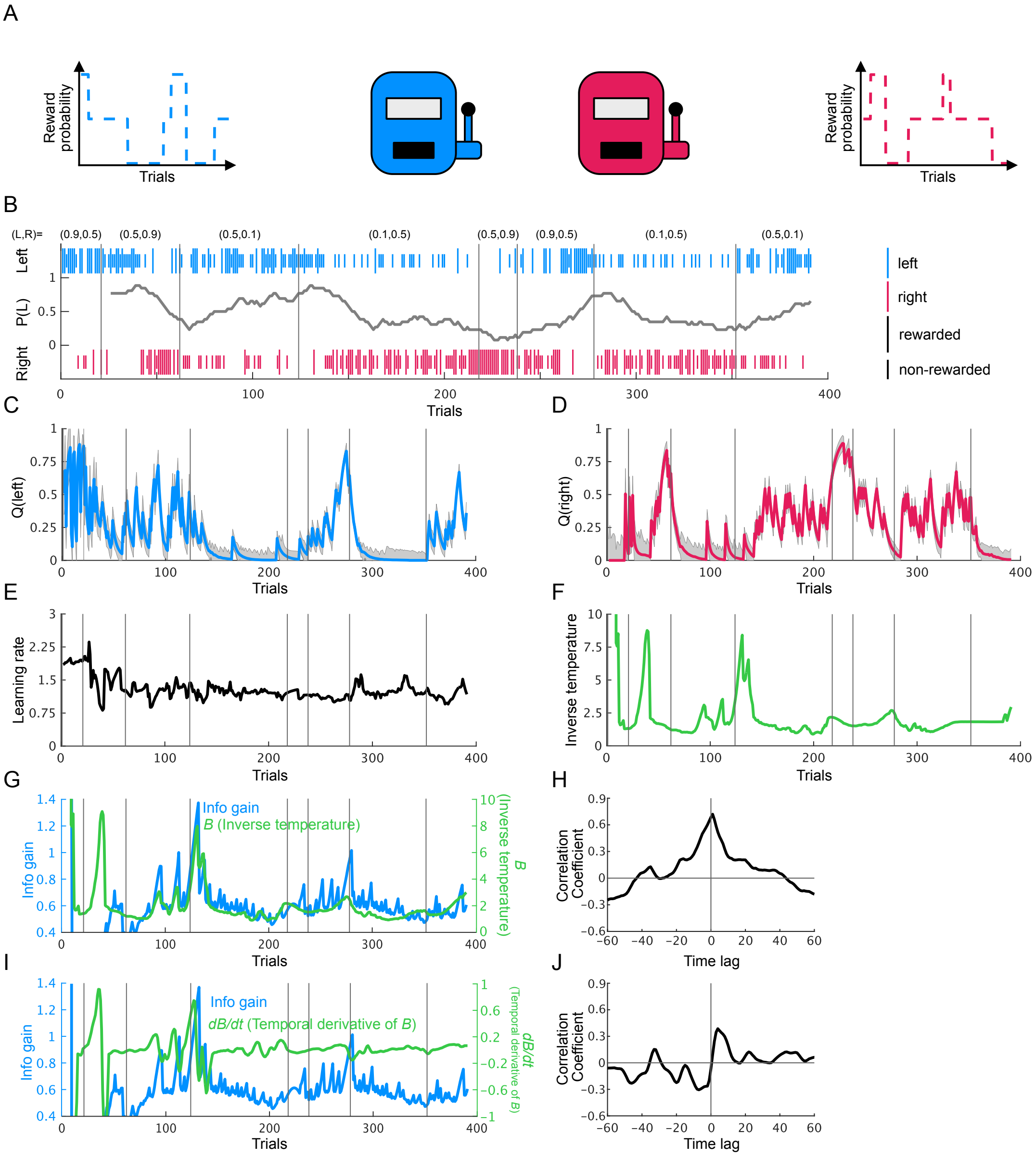

Supplementary Fig. 7: Estimation of the rat's internal state by inverse Q-learning

(A, B) The same as Fig. 5. **(C, D)** Rat behavioral data-driven estimations of action value functions Q for left (C) and right (D) options. Continuous shaded error bars represent standard deviations for all particles. **(E, F)** Rat behavior-driven estimation of agent's learning rate α (E) and inverse temperature β (F). **(G, H)** Time-series of the intensity of inverse temperature and the sum of the expected information gains for both options (G), and their cross-correlation (H). **(I, J)** Time-series of the temporal derivative of inverse temperature and the sum of the expected information gains for both options (I), and their cross-correlation (J). The temporal derivative was computed by linear regression within time window of seven trials. Number of particles was 100,000 in the particle filter.

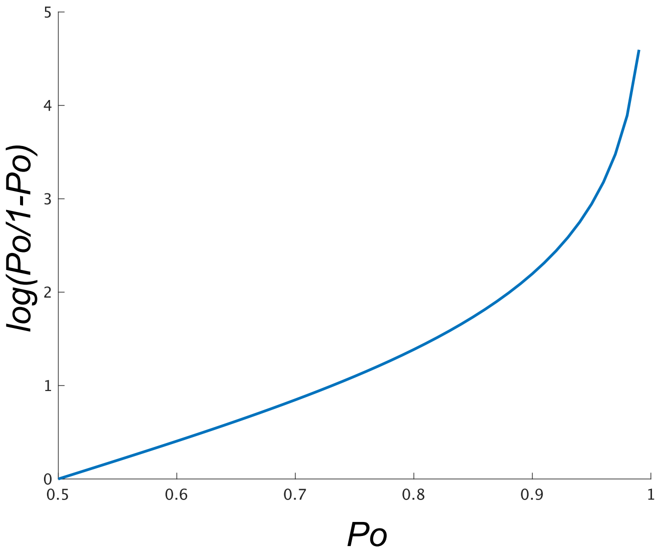

Supplementary Fig. 8: Logit function for reward

Reward profile represented by Logit function of P_o.
