# Supplementary material for: Decoding reward–curiosity conflict in decision-making from irrational behaviors
Source: Nat Comput Sci. 2023 May 15;3(5):418–32. doi: 10.1038/s43588-023-00439-w (PMC10768639; doi:10.1038/s43588-023-00439-w)
Supplement: Supplementary file 2 — Reporting Summary [file 43588_2023_439_MOESM2_ESM.pdf]

nature research

Corresponding author(s): Honda Naoki

Last updated by author(s): March 29,2023

Reporting Summary

Nature Research wishes to improve the reproducibility of the work that we publish. This form provides structure for consistency and transparency in reporting. For further information on Nature Research policies, see our Editorial Policies and the Editorial Policy Checklist.

Statistics

For all statistical analyses, confirm that the following items are present in the figure legend, table legend, main text, or Methods section.

n/a	Confirmed	
☐	☒	The exact sample size (*n*) for each experimental group/condition, given as a discrete number and unit of measurement
☒	☐	A statement on whether measurements were taken from distinct samples or whether the same sample was measured repeatedly
☐	☒	The statistical test(s) used AND whether they are one- or two-sided<br>*Only common tests should be described solely by name; describe more complex techniques in the Methods section.*
☒	☐	A description of all covariates tested
☐	☒	A description of any assumptions or corrections, such as tests of normality and adjustment for multiple comparisons
☐	☒	A full description of the statistical parameters including central tendency (e.g. means) or other basic estimates (e.g. regression coefficient) AND variation (e.g. standard deviation) or associated estimates of uncertainty (e.g. confidence intervals)
☐	☒	For null hypothesis testing, the test statistic (e.g. F, t, r) with confidence intervals, effect sizes, degrees of freedom and P value noted<br>*Give P values as exact values whenever suitable.*
☐	☒	For Bayesian analysis, information on the choice of priors and Markov chain Monte Carlo settings
☒	☐	For hierarchical and complex designs, identification of the appropriate level for tests and full reporting of outcomes
☐	☒	Estimates of effect sizes (e.g. Cohen's d, Pearson's r), indicating how they were calculated

Our web collection on statistics for biologists contains articles on many of the points above.

Software and code

Policy information about availability of computer code

Data collection	No software was used for data collection
Data analysis	The computer simulation and data analysis were done using Matlab software (Version R2020b). The code used for this work are available on GitHub at: https://github.com/YukiKonaka/Konaka_Honda_2023. The specific version used to produce the results in this manuscript is also available on Zenodo at https://doi.org/10.5281/zenodo.7722905.<br><br>The algorithm used in this study is particle filter in control system toolbox (Version R2020b). We also used shaded ErrorBar (GNU LESSER GENERAL PUBLIC LICENSE Version 3, 29 June 2007), which is public MATLAB function located at https://github.com/raacampbell/shadedErrorBar/blob/master/shadedErrorBar.m. The file of shaded ErrorBar was also uploaded at our GitHub (https://github.com/YukiKonaka/Konaka_Honda_2023) and our Zenodo (https://doi.org/10.5281/zenodo.7722905).

For manuscripts utilizing custom algorithms or software that are central to the research but not yet described in published literature, software must be made available to editors and reviewers. We strongly encourage code deposition in a community repository (e.g. GitHub). See the Nature Research guidelines for submitting code & software for further information.

Data

Policy information about availability of data

All manuscripts must include a data availability statement. This statement should provide the following information, where applicable:

- Accession codes, unique identifiers, or web links for publicly available datasets
- A list of figures that have associated raw data
- A description of any restrictions on data availability

Source data for figures 2, 3, 5 and 6 are available with this paper. Source data for Supplementary Figures are available in Supplementary Data. We used the rat

behavioral data published by Makoto Ito and Kenji Doya, Validation of Decision-Making Models and Analysis of Decision Variables in the Rat Basal Ganglia, Journal of Neuroscience (Publisher: the Society for Neuroscience), 2009 (DOI: 10.1523/JNEUROSCI.6157-08.2009), which is publicly available at Prof. Doya Kenji's homepage: https://groups.oist.jp/ja/ncu/data. This rat behavioral data is also included in Source data for figure 5 and Zenodo at https://doi.org/10.5281/zenodo.7722905.

Field-specific reporting

Please select the one below that is the best fit for your research. If you are not sure, read the appropriate sections before making your selection.

☒ Life sciences ☐ Behavioural & social sciences ☐ Ecological, evolutionary & environmental sciences

For a reference copy of the document with all sections, see nature.com/documents/nr-reporting-summary-flat.pdf

Life sciences study design

All studies must disclose on these points even when the disclosure is negative.

Sample size	Data size is based on the rat behavioral data from Ito & Doya Journal of Neuroscience 2009, which was cited in the manuscript.
Data exclusions	In the rat data of the two-choice task (Ito & Doya Journal of Neuroscience 2009), a rat selected left or right, but sometimes fail to select. In our analysis, we excluded no-choice trials from the behavioral time-series because we assumed the rat cannot update the recognition because of no observation of reward in the no-choice trials.
Replication	We checked the estimation performance by replicating the estimations (Supplementary Fig. 3).
Randomization	This is not relevant to our study because we only decipher the temporal dynamics of the internal state including curiosity meta-parameter from the datasets. There is not allocation procedure.
Blinding	No data collection was involved in the present study. Blinding was not possible because we only decode curiosity from the datasets, there is no allocation procedure.

Reporting for specific materials, systems and methods

We require information from authors about some types of materials, experimental systems and methods used in many studies. Here, indicate whether each material, system or method listed is relevant to your study. If you are not sure if a list item applies to your research, read the appropriate section before selecting a response.

Materials & experimental systems

n/a	Involved in the study	
☒	☐	Antibodies
☒	☐	Eukaryotic cell lines
☒	☐	Palaeontology and archaeology
☒	☐	Animals and other organisms
☒	☐	Human research participants
☒	☐	Clinical data
☒	☐	Dual use research of concern

Methods

n/a	Involved in the study	
☒	☐	ChIP-seq
☒	☐	Flow cytometry
☒	☐	MRI-based neuroimaging
